# Phosphorylation of LSD1 by PLK1 promotes its chromatin release during mitosis

**DOI:** 10.1186/s13578-017-0142-x

**Published:** 2017-03-23

**Authors:** Bin Peng, Ruifeng Shi, Weiwei Jiang, Yue-He Ding, Meng-Qiu Dong, Wei-Guo Zhu, Xingzhi Xu

**Affiliations:** 1 Beijing Key Laboratory of DNA Damage Response and College of Life Sciences, Capital Normal University, Beijing, 100048 China; 2 Guangdong Key Laboratory of Genome Stability & Disease Prevention, Shenzhen University School of Medicine, Shenzhen, 518060 Guangdong China; 3 National Institute of Biological Sciences, Beijing, 102206 China

**Keywords:** PLK1, Lysine-specific demethylase LSD1, Phosphorylation, Mitosis, Chromatin release

## Abstract

**Background:**

Lysine-specific histone demethylase 1 (LSD1) modulates chromatin status through demethylation of H3K4 and H3K9. It has been demonstrated that LSD1 is hyperphosphorylated and dissociates from chromatin during mitosis. However, the molecular mechanism of LSD1 detachment is unknown.

**Results:**

In this report, we found that polo-like kinase 1 (PLK1) directly interacted with LSD1 and phosphorylated LSD1 at Ser-126 . Nocodazole-induced metaphase arrest promoted release of LSD1 from chromatin, and the phosphorylation-defective mutant LSD1 (S126A) failed to dissociate from chromatin upon nocodazole treatment.

**Conclusions:**

Taken together, our findings demonstrate that phosphorylation of LSD1 at Ser-126 by PLK1 promotes its release from chromatin during mitosis.

## Background

Histone post-translational modifications (PTMs) serve as major regulatory mechanisms for chromatin structure and function [1]. While the presence of methylation and acetylation [2] and phosphorylation [3] on histones was described about half a century ago, many more PTMs have been demonstrated to occur on histones [4]. Given that chromatin dynamics plays an essential role both in maintenance of cellular homeostasis and in cellular stress responses, histone PTMs have been implied in most, if not all physiological and pathological processes. However, the biological significance and the molecular mechanism of these PTMs, particularly the crosstalk and joint effects of different PTMs on the same molecule, are largely elusive, and deciphering the regulatory networks of the protein machineries that incorporate (write), remove (erase), and bind (read) histone PTMs is of high interest.

Lysine-specific demethylase 1 (LSD1), a flavin adenine dinucleotide (FAD)-dependent amine oxidase, was the first identified demethylase (eraser) for lysine methylation of histones and non-histone proteins [5]. It specifically removes methyl groups via a redox process of mono- or di-methylated histone H3 lysine4 (H3K4) [6] and H3 lysine 9 (H3K9) [7]. LSD1 in the CoREST-HDAC containing repressor complexes, functions as a corepressor by mediating demethylation of H3K4me [6]. On the other hand, LSD1 is recruited to the promoter regions of androgen receptor (AR) target genes and demethylates H3K9me, co-activating AR-dependent transcription [7]. Misregulated expression of LSD1 has been reported in several cancer types [5, 8].

Our lab reported previously that LSD1 is hyperphosphorylated in response to nocodazole-induced metaphase arrest [9]. It was demonstrated recently that LSD1 dissociates from chromatin during mitosis [10]. We thus sought to uncover a potential link between LSD1 and mitotic kinases. Polo-like kinase 1 (PLK1), a major mitotic serine/threonine protein kinase, regulates several events throughout M phase of the cell cycle, from mitotic entry to mitotic exit and cytokinesis [11]. Its functions are executed by binding and phosphorylating proteins through its polo-box domain and kinase domain, respectively. In this report, we found that PLK1 interacts with and phosphorylates LSD1 at Ser126 and this phosphorylation promotes LSD1 release from chromatin during mitosis.

## Results and discussion

### LSD1 directly interacts with PLK1

**Fig. 1:**
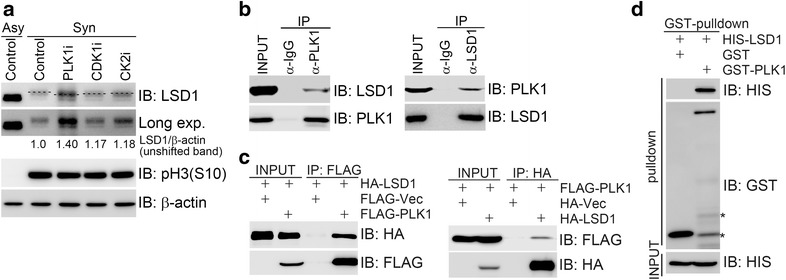
LSD1 directly interacts with PLK1. **a** PLK1 inhibitor treatment reduced thymidine-nocodazole-induced LSD1 mobility shift in HeLa cells. HeLa cells were synchronized at metaphase by single thymidine block followed by nocodazole treatment for 12 h. Kinase inhibitors were added 2 h before total cell lysates were harvested. A *dash line* indicates the center of the shifted band of LSD1. Relative ratio of the unshifted band intensity of LSD1/β-actin was presented. Asy: asynchronized cells; syn: synchronized cells; long exp.: long exposure. **b** Endogenous LSD1 interacts with PLK1. Total cell lysates from 293T cells were subjected to immunoprecipitation followed by immunoblotting with antibodies as indicated. **c** Ectopic-tagged LSD1 interacts with PLK1. FLAG-PLK1 and HA-LSD1 were co-expressed in 293T cells. Total cell lysates were harvested 48 h after transfection and subjected to immunoprecipitation followed by immunoblotting with anti-HA or anti-FLAG antibodies. **d** LSD1 directly interacts with PLK1. Bacterially produced GST-PLK1 was used to pull down bacterially produced HIS-LSD1. *Asterisk* non-specific signal

Our laboratory and others demonstrated previously that LSD1 was hyperphosphorylated during mitosis [9]. The major mitotic protein kinases include polo-like kinase 1 (PLK1), CDK1, and CK2. We thus employed inhibitors of theses kinases to treat thymidine-nocodazole synchronized HeLa cells (Fig. 1a). We found that PLK1 inhibitor treatment, and to a lesser extent, CDK1 inhibitor treatment, reduced thymidine-nocodazole treatment-induced retarded migration of LSD1, while CK2 inhibitor treatment did not have such an effect (Fig. 1a). This indicates that PLK1 could be one of the kinases for LSD1 phosphorylation during mitosis. To confirm this, we performed co-immunoprecipitation assays to examine the potential interaction between PLK1 and LSD1. It was found that endogenous LSD1 was present in the endogenous PLK1 immunocomplex in 293T cells and vice versa (Fig. 1b). To exclude the possibility that this interaction was derived from antibody cross-reactivity, HA-LSD1 and FLAG-PLK1 were co-expressed in 293T cells; subsequent co-immunoprecipitation assays revealed that HA-LSD1 was present in the anti-FLAG immunocomplex and vice versa (Fig. 1c). Furthermore, bacterially produced GST-PLK1 was able to pull down bacterially produced HIS-LSD1 (Fig. 1d). Taken together, these results demonstrated that PLK1 directly interacts with LSD1.

**Fig. 2:**
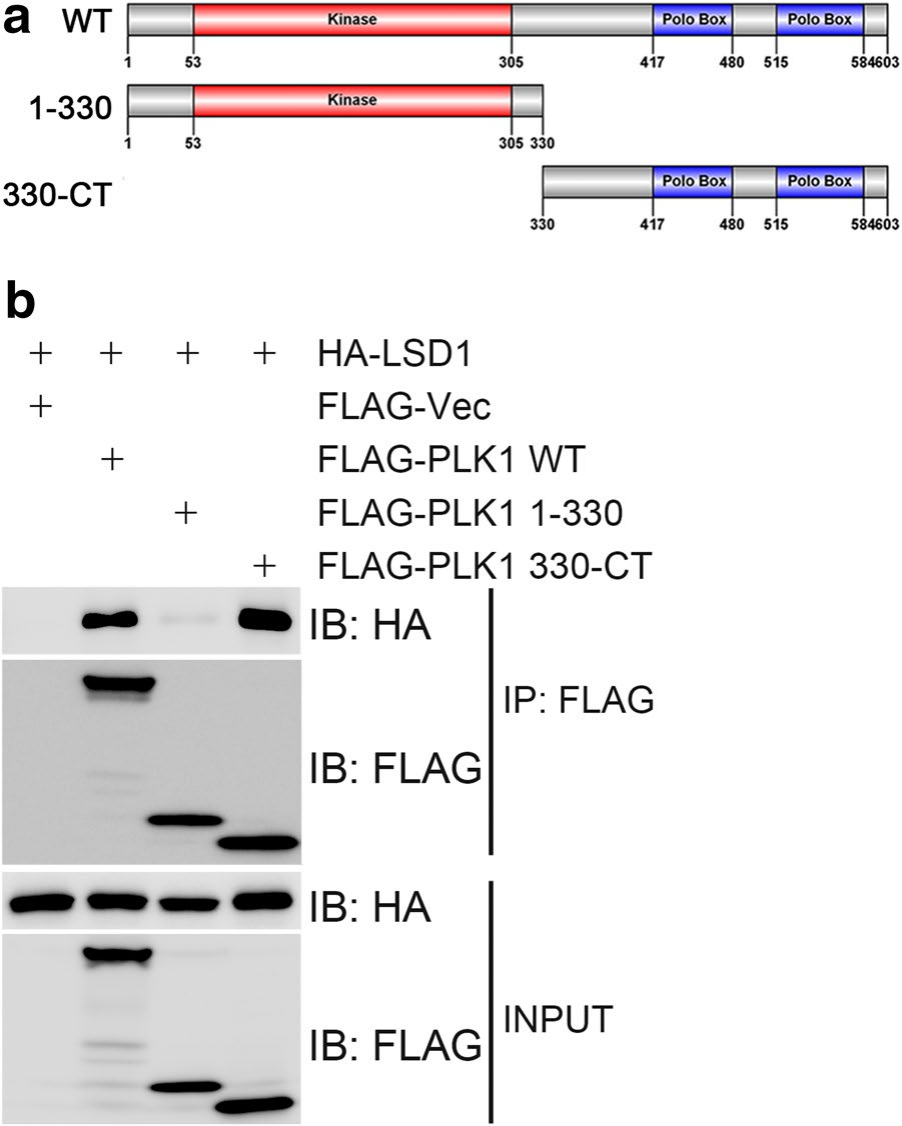
The Polo-box domains of PLK1 mediate its interaction with LSD1. **a** Domain structure of PLK1. *PLK1* encodes a polypeptide of 603 AAs. Its amino terminus (1-330 AAs) contains the kinase domain, while its carboxyl terminus (330-CT) harbors two Polo boxes. **b** Carboxyl terminus of PLK1 mediated its interaction with LSD1. HA-LSD1 and wild-type FLAG-PLK1 or its truncation mutants were transiently co-expressed in 293T cells, total cell lysates were harvested 48 h after transfection and subjected to immunoprecipitation followed by immunoblotting with antibodies as indicated

**Fig. 3:**
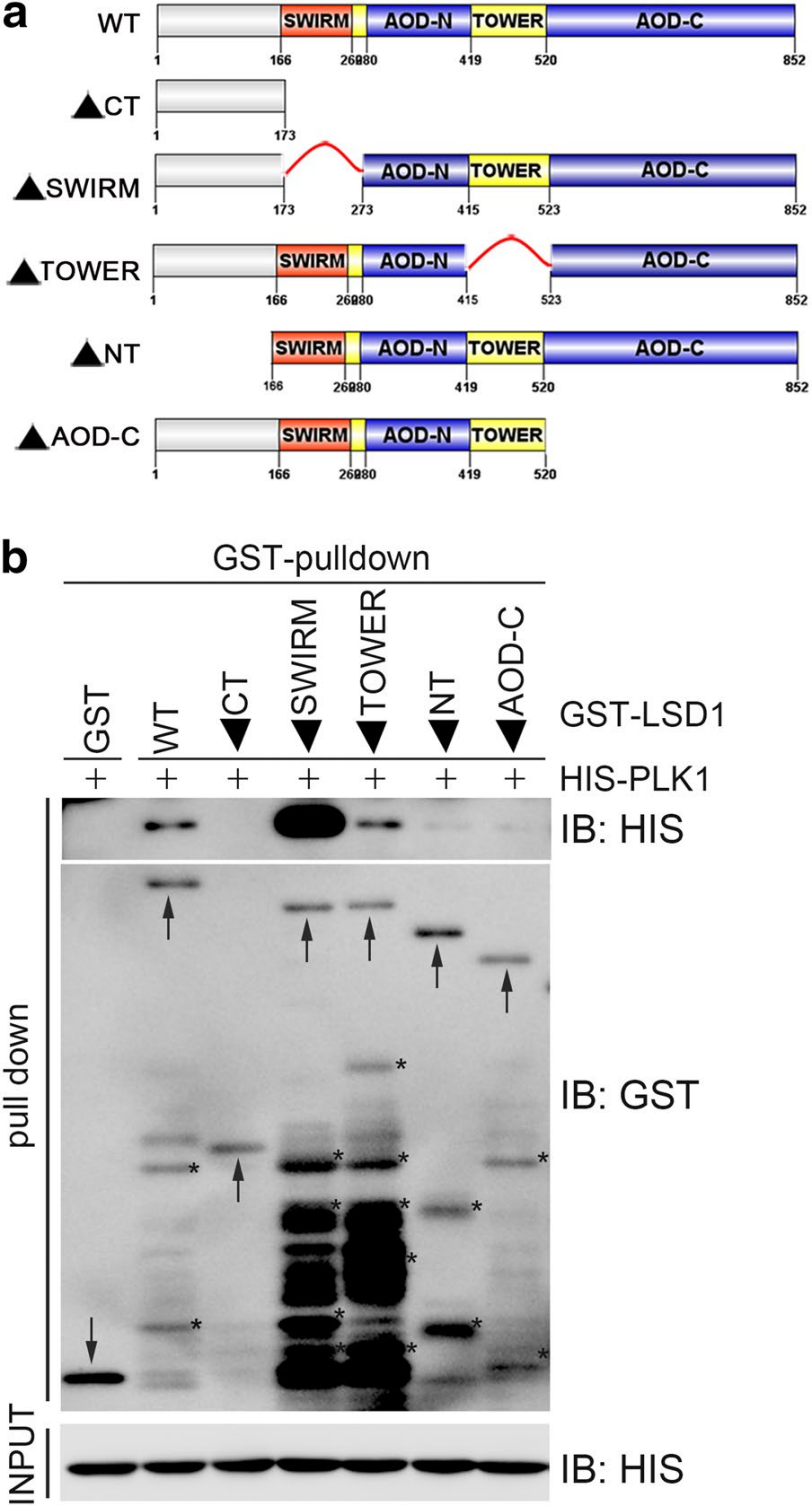
Both amino terminus and carboxyl terminus of LSD1 are important for its interaction with PLK1. **a** Schematic diagram of LSD1. *LSD1* encodes a polypeptide of 852 AAs with a SWIRM domain, a TOWER domain in between an amino amine oxidase (AOD) domain (AOD-N) and a carboxyl AOD domain (AOD-C). *NT* amino terminus; *CT* carboxyl terminus. **b** Both NT and CT of LSD1 are important for its direct interaction with PLK1. Bacterially produced GST fusions of LSD1 and its deletion mutants were used to pull down bacterially produced HIS-PLK1. An *arrow points* to the target protein

To further dissect this interaction, we generated two PLK1 truncation mutants, namely FLAG-PLK1(1-330) that harbors the kinase domain and FLAG-PLK1(330-CT) that contains two polo box domains (Fig. 2a). It was found that HA-LSD1 was present in the FLAG-PLK1 and FLAG-PLK1(330-CT) immunocomplexes, but not in the FLAG-PLK1(1-330) immunocomplex (Fig. 2b). We also generated several deletion mutants of LSD1 with a GST epitope (Fig. 3a), and these mutants were bacterially expressed, affinity-purified, and applied for GST pulldown assays with bacterially produced HIS-PLK1 (Fig. 3b). It was found that both the mutant with deletion of the amino terminus of LSD1 (1-165 aas) and the mutant with deletion of the carboxyl terminal AOD failed to pull down HIS-PLK1, while the mutant with deletion of the SWIRM domain enhanced the pulldown of HIS-PLK1 (Fig. 3b). This indicates that both the amino terminus and the carboxyl AOD are required for the interaction between PLK1 and LSD1.

### PLK1 phosphorylates LSD1 at Ser-126

**Fig. 4:**
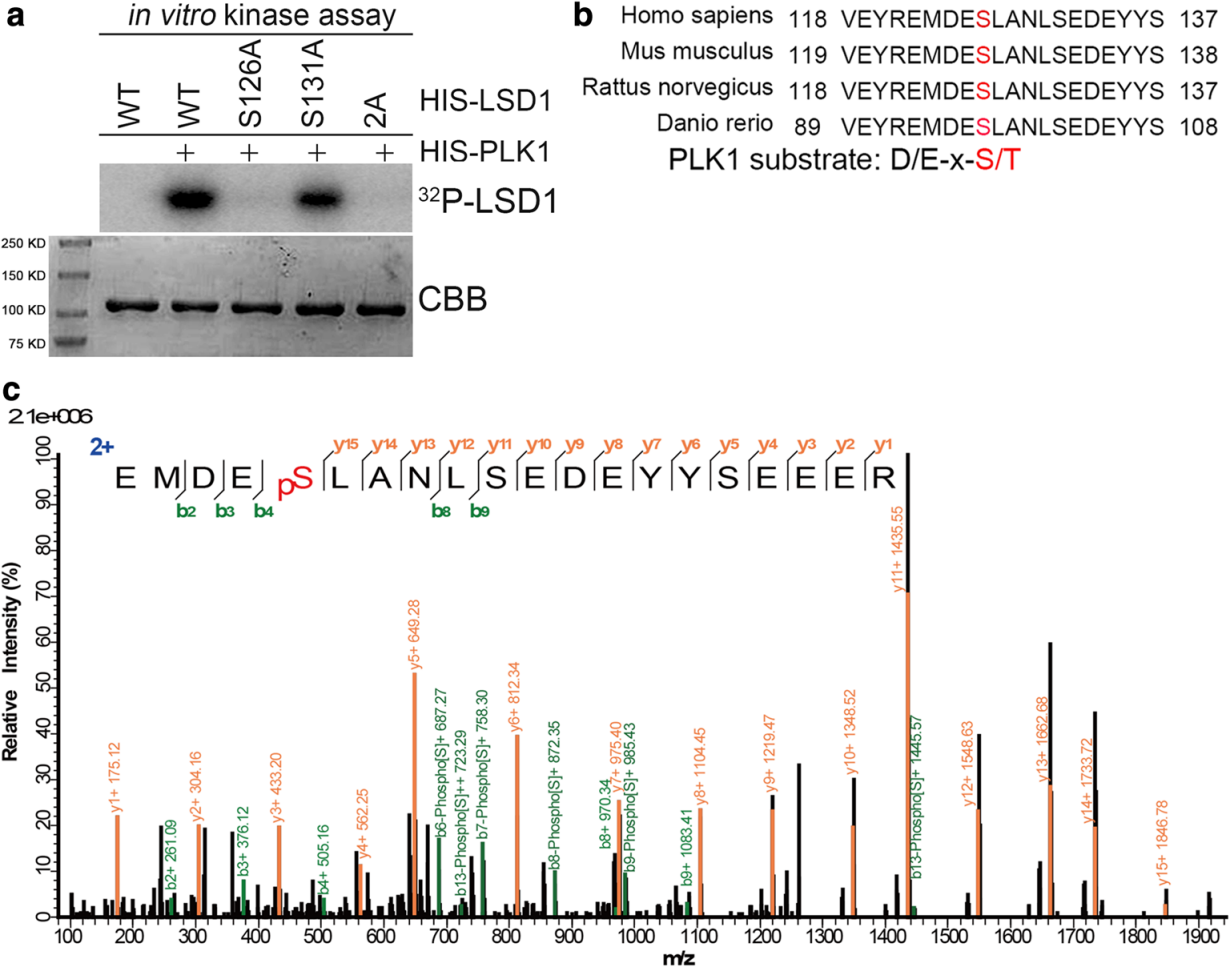
PLK1 phosphorylates LSD1 at Ser-126. **a** PLK1 phosphorylates LSD1 in vitro. In vitro kinase assays in the presence of ^32^P-ATP were performed by incubating bacterially produced HIS-PLK1 with HIS-LSD1 or its point mutants. **b** Mass spectrometry analysis identified Ser126 as the confident phosphorylation sites of LSD1 by PLK1. In vitro phosphorylated LSD1 by PLK1 with cold ATP was subjected to trypsin digestion and mass spectrometric analysis. **c** Ser126 of human LSD1 is conserved from human to zebrafish. Polypeptides surrounding the Ser-126 site from different species were aligned

Given that LSD1 is hyperphosphorylated upon nocodazole-induced metaphase arrest [9] and that LSD1 directly associates with PLK1 (Fig. 1), we reasoned that LSD1 could be a substrate of PLK1. *In vitro* kinase assays unveiled that bacterially produced PLK1 efficiently incorporated radiolabeled phosphorus-32 into bacterially produced HIS-LSD1 (Fig. 4a). In vitro phosphorylated LSD1 was subjected to trypsin digestion and subsequent mass spectrometric analysis, by which a single phosphorylation site, namely Ser-126 on LSD1 was identified (Fig. 4b). Consistent with the mass spectrometric analysis result, mutation of Ser-126 to Ala fully abolished PLK1-mediated phosphorylation on LSD1, while mutation of the adjacent Ser-131 to Ala, which is a CK2-mediated phosphorylation site [12], did not have a similar effect (Fig. 4a). Sequence alignment of LSD1 from different species from human to zebrafish demonstrated that human LSD1 Ser-126 residue is well conserved (Fig. 4c), implying this phosphorylation site could be functionally relevant. To examine if this phosphorylation event occurs in vivo, we attempted twice to generate phosphorylation-specific LSD1(S126) antibody. However, the affinity-purified IgG failed to detect endogenous phosphorylated form of LSD1, though it was reactive to in vitro phosphorylated form of LSD1 by PLK1 (data not shown). Taken together, our results demonstrated that LSD1 is phosphorylated by PLK1 at Ser-126, and this phosphorylation site is conserved in different species.

### Phosphorylation of LSD1 by PLK1 promotes its release from chromatin during mitosis

**Fig. 5:**
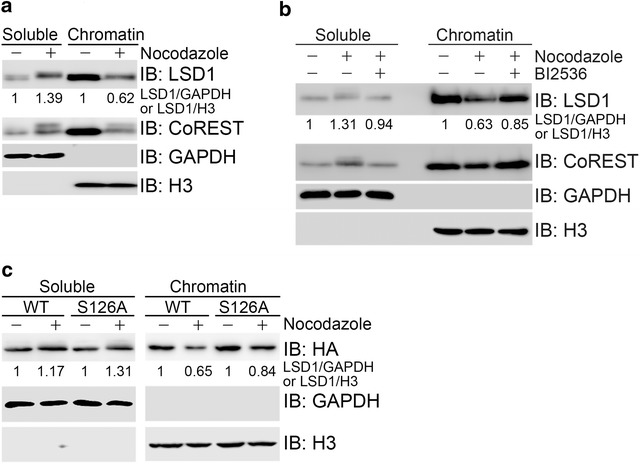
Phosphorylation of LSD1 by PLK1 promotes its chromatin release during mitosis. **a** Nocodazole treatment induced LSD1 release from chromatin. HeLa cells were asynchronized or synchronized at metaphase by single thymidine block followed by nocodazole treatment for 12 h and then subjected to chromatin fractionation. *Soluble* cytosol and nuclear soluble fraction, *chromatin* chromatin-enriched fraction. Relative ration of the band intensity of total LSD1/GAPDH in the soluble fraction or total LSD1/H3 in the chromatin-enriched fraction was presented in (**b**) and (**c**) as well. **b** PLK1 inhibitor treatment blocked nocodazole-induced chromatin release of LSD1. HeLa cells were prepared as in **a** except that a fraction of thymidine-nocodazole synchronized cells were treated with BI2536, a PLK1 inhibitor, 2 h before harvest. **c** Phosphorylation-defective mutant LSD1 (S126A) failed to release from chromatin during mitosis. HEK293 cells stably expressing HA-LSD1 or HA-LSD1(S126A) were subjected to chromatin fractionation

We then sought to determine the functional significance of PLK1-mediated phosphorylation of LSD1 at Ser-126. It has been demonstrated that H3K9me3 dramatically increases in G2 to reach a maximum at metaphase, maintaining pericentric heterochromatin [13–15]. LSD1 seems to work with androgen receptor (AR) toward demethylation of H3K9me1/2 [7], while JMJD2C specifically demethylates H3K9me3 [16]. It was further demonstrated that AR, JMJD2C and LSD1 jointly assemble on chromatin to remove methyl groups from mono, di and trimethylated H3K9 [17]. It was also reported that LSD1 dissociates from chromatin during mitosis in embryonic stem cells [10]. Therefore, we speculated that LSD1 would be released from chromatin upon nocodazole-induced arrest at metaphase in cultured cells. Indeed, chromatin fractionation assays found that protein levels of LSD1 and its partner CoREST in chromatin-enriched fraction declined upon nocodazole treatment (Fig. 5a), and this decrease was restored upon pretreatment with BI2536, an inhibitor of PLK1 kinase (Fig. 5b). Furthermore, HA-LSD1, but not the phosphorylation-defective mutant HA-LSD1(S126A), was reduced in the chromatin enriched fraction upon nocodazole treatment (Fig. 5c). It was noted that LSD1 retained in the chromatin-enriched fraction in response to nocodazole treatment was hypophosphorylated, while it was hyperphosphorylated in the nuclear soluble fraction (Fig. 5a, b). These findings demonstrate that PLK1-mediated phosphorylation of LSD1 at Ser-126 promotes LSD1 release from chromatin during mitosis.

Histone methylation at specific lysine residues plays a critical role in regulating chromatin structure and gene expression and subsequent cellular activities. Fine control of the balance of histone methyltransferase/demethylase activities is essential for cell cycle progression and maintenance of genome integrity [18]. Tipping the balance toward either direction could be detrimental. For example, LSD1 serves for demethylation of H3K4 and H3K9 [5], its gene deletion was reported in pancreatic ductal adenocarcinoma (7/109 = 6.4%) [19], while its gene amplification was found in neuroendocrine prostate cancer (6/107 = 5.6%) (http://www.cbioportal.org/) and sarcoma (6/207 = 2.9%) [20]. LSD1 RNA expression levels vary a lot in different cancer types (http://www.cbioportal.org/). These findings imply that a delicate control of LSD1 expression levels, subcellular localization, and enzymatic activity may be a requirement for genome stability. Whole-genome mapping demonstrated that LSD1 genomic regions overlaps with H3K4me2 genomic regions and both are enriched on the promoters of highly expressed genes in ES cells [10], while H3K9me3 levels peak at the pericentromeric regions during mitosis [13–15]. To reduce general transcriptional activity to a minimum and maintain high H3K9me3 levels at the pericentromeric regions during mitosis, histone demethylase activities on chromatin should be reduced.

## Conclusions

Our study has uncovered a novel regulatory mechanism for controlling histone demethylase activity, in which PLK1-mediated phosphorylation of LSD1 during mitosis expels it from chromatin.

## Methods

### Cell cultures, reagents, antibodies

HeLa and HEK293T cells were grown in high-glucose Dulbecco’s Modified Eagle’s Medium (DMEM) supplemented with 10% fetal bovine serum at a 37 °C incubator with 5% CO_2_.

Nocodazole (M1404, a final concentration of 340 nM was used throughout this research) and Thymidine (T1895, a final concentration of 2 mM was used) were purchased from Sigma. BI2536, a PLK1 inhibitor (S1109, a final concentration of 1 μM was used), was purchased from Selleck.

Rabbit polyclonal antibodies used for immunoblotting and immunoprecipitation in this study including anti-HA (A190–208A), anti-LSD1 (A300–215A), anti-CoREST (A300–130A), anti-GAPDH (A300–643A) and anti-H3 Ser10 (A301–844A) were from Bethyl Laboratories. Rabbit polyclonal anti-H3 antibody (#9715) was from Cell Signaling Technology. Mouse monoclonal antibody against GST (A00865) was from GenScript Corporation. Mouse monoclonal anti-FLAG M2 (F1804) was from Sigma. Mouse monoclonal anti-HIS (D291-3) was from MBL Biotech.

### Expression constructs

Human cDNA clones encoding full length and deletion mutants of LSD1, PLK1 were subcloned into pcDNA3.0 with three copies of HA or FLAG epitope at its N-terminus for expression in mammalian cells or into pET28(A) or pGEX-4T-1 for producing HIS or GST recombinant fusion protein in *E. coli*. Point mutants (LSD1(S126A), LSD1(S131A), and LSD1(2A)) were generated using the QuikChange mutagenesis kit (SBS Genetech Co).

### Immunoblotting and immunoprecipitation

Both immunoblotting and immunoprecipitation assays were performed with desired antibodies according to the protocols described before [12, 21].

### GST-pulldown and in vitro kinase assay

Bacterially-purified GST fusions (1 μg) were incubated with bacterially produced HIS tagged fusions (1 μg) in 500 μl of NETN buffer at 4 °C overnight. Glutathione-Sepharose beads (20 μl per pulldown) were added and incubated for 1 h before extensively washing with NETN buffer.

For in vitro kinase assays [12, 22], bacterially-purified HIS tagged PLK1 and LSD1 were incubated at kinase buffer containing 50 mM HEPES (pH 7.4), 10 mM MgCl_2_, 1 mM DTT, 1 mM Na_3_VO_4_, 10 μM coldATP and 5 μCi [γ-^32^P]-ATP at 30 °C for 30 min.

### Identification of phosphorylation sites of LSD1 by PLK1 using mass spectrometry

Phosphorylation site mapping by mass spectrometry was performed as described previously [23]. Briefly, in vitro phosphorylated proteins were precipitated with TCA and resuspended in a buffer containing 8 M urea, 100 mM Tris, pH8.5. After reduction and alkylation, the sample was digested by Trypsin overnight at 37 °C. The peptides were analyzed on an Easy-nLC 1000 UPLC (Thermo Fisher Scientific) coupled to a Q exactive mass spectrometer (Thermo Fisher Scientific). Peptides were loaded on a pre-column (75 μm ID and packed with 8 cm ODS-AQ 12 nM S-10 mm (YMC Co., Ltd)) and separated on an analytical column (75 μm ID and packed with 11 cm Luna 3 μm 100Å resin (Phenomenex)) with an acetonitrile gradient from 0 to 30% in 55 min and 30–80% in another 10 min at a flow rate of 300 nl/min. Spectra were acquired in a data-dependent mode: the 10 most intense ions except charge 1+ or unassigned from each full scan (Resolution 70,000) were isolated for HCD MS2 (Resolution 17,500) at NEC 27 with a dynamic exclusion time of 60 s. For peptide identification, the MS2 spectra were searched against an artificial database (including GST-LSD1 and HIS-PLK1 sequences in a *C. elegans* WS217 database) using Prolucid [24]. Search results were filtered using DTASelect 2.0 [25] with 7 p.p.m. mass accuracy for precursor mass and a 5% FDR cutoff. The phosphorylation spectra presented in the figures were annotated using pLabel [26].

### Chromatin fractionation and cell synchronization

Chromatin fractionation was performed essentially as described before [21]. HeLa and HEK293 cells were synchronized at metaphase by thymidine-nocodazole block as described before [27].

## Abbreviations

CoREST: corepressor for element silencing transcription factor
CDK1: cyclin-dependent kinase 1
CK2: casein kinase II
H3K4: histone H3 lysine 4
H3K9: histone H3 lysine 9
LSD1: lysine-specific demethylase 1
PLK1: polo-like kinase 1
PTM: post-translational modification
